# Outcome in patients with enthesitis related arthritis (ERA): juvenile arthritis damage index (JADI) and functional status

**DOI:** 10.1186/1546-0096-6-18

**Published:** 2008-10-22

**Authors:** Pradip Kumar Sarma, Ramnath Misra, Amita Aggarwal

**Affiliations:** 1 Department of Immunology, Sanjay Gandhi Postgraduate Institute of Medical Sciences, Lucknow, India

## Abstract

**Background:**

Data on outcome of ERA is scarce and there is lack of well-accepted tools. JADI is a newly described outcome measure in JIA that has not been evaluated in ERA. We studied outcome in ERA using JADI and correlated it with traditional outcome measures.

**Methods:**

We studied 49 consecutive patients of ERA with age ≥ 5 years and duration ≥ 1 year. Along with JADI, we recorded enthesitis, lumbar spinal anterior flexion by modified Schober's method, presence of inflammatory backache, loss of school years, HAQ-S, growth and pubertal delay. Parent's/patient's and physician's global assessments on 100 mm visual analogue scale.

**Results:**

The median age was 18.0 (10–27) years and the median duration of disease was 6.0 (1–17) years. All the patients were male and half (53.1%) were HLA B 27 positive. Fourteen had decreased anterior lumbar flexion movement and 32 had inflammatory backache. Active enthesitis was present in 63.3%. Functionally, mild, moderate and severe disability was seen in 18.4%, 34.7% & 14.3% respectively. Sixty five percent of patients lost education years. Twenty-eight patients had damaged joints with median of 2.0 joints (0–9). Seventeen patients (34.7%) had damaged joints in JADI-A score with a median of 1.0 (0–12). Growth failure was the commonest extra articular damage (8.2%) in JADI-E. JADI correlated with HAQ-S, parent's or patient's & physician's global assessment (p < 0.01). Limitation of spinal mobility had high correlation with HAQ-S; correlation with JADI-A was low.

**Conclusion:**

Three fourth of the ERA patients had functional limitations. Half of the patients had damaged joints. Even though JADI correlated well with traditional outcome measures, it underestimates joint damage, and does not assess enthesitis and spinal limitation which affect functional status in ERA. Inclusion of these may make it more useful for ERA.

## Introduction

Enthesitis-related arthritis (ERA), as defined by ILAR criteria [1], is characterized by the involvement of the entheses and the axial skeleton along with peripheral joints. It is an HLA B-27 related disease and is similar to traditionally defined juvenile spondylarthropathy, including juvenile ankylosing spondylitis (JAS), seronegative enthesopathy and arthropathy (SEA) syndrome, and undifferentiated juvenile spondylarthropathy, though there is controversy regarding use of various terminology [2-5]. Although adult-onset AS (AoAS) and JAS share many common features, JAS is associated with worse functional outcome compared with adult onset ankylosing spondylitis [6]. Spinal deformity is also more common in JAS than adult onset AS [6].

In most outcome studies in Juvenile Idiopathic Arthritis (JIA), number of patients with ERA is small compared to other subtypes of JIA. Remission rates in ERA have ranged from 17% to 37% and the frequency of severe disability from 4% to 52% [7-11]. In a recently published study where ILAR criteria was used for case definition, patients with ERA had lower levels of physical functioning, poorer physical health, and more bodily pain compared to patients with oligoarthritis or polyarthritis after a median disease duration of 15.3 years [12].

There is no universally accepted outcome measure for ERA patients. Many use development of radiological sacroiliitis or ankylosing spondylitis as an outcome measure; however, radiology is not suitable for as radiological sacroilitis may take decades to develop. Radiological indices developed for other subtypes of JIA do not reflect true damage in ERA as such indices measure damages in wrists and knees, whereas in ERA involvement of hip joint and spine are more common.

Childhood health Assessment Questionnaire (CHAQ) is the most commonly used tool to assess physical disability due to disease. CHAQ evaluates physical functions in dressing, getting up, walking, eating, hygiene, reaching overhead objects, grip and activities. In ERA, spine is also involved in contrast to other subtypes of JIA and CHAQ does not contain items to measure functional limitation because of spinal involvement. HAQ modified for spondyloarthropathy (HAQ-S) has been used to measure functional outcome in young adults with ERA [14,12]. In the HAQ-S, 5 items forming 2 areas, bending and driving, are added to the HAQ [14]. HAQ-S has been evaluated in adult patients with spondyloarthropathy [14].

The Juvenile Arthritis Damage Index (JADI) is a newly described outcome measure in JIA [13]. It assess overall articular (JADI-A) and extraarticular damage (JADI-E). The JADI-A score had high correlation with the number of joints with limited range of motion and moderate correlation with the childhood HAQ (CHAQ), Steinbrocker functional classification, and radiographic damage. The JADI-A also performed well to differentiate different levels of disability. The patients with ERA were excluded from this study.

Though according to the western literature 11–16% of patients with JIA have ERA [2-5,12], in India it is probably the commonest subtype of JIA seen by rheumatologists [15]. Data on outcome of ERA patients from India are scarce. Therefore, we studied the outcome of ERA subtypes with both traditional outcome measures and JADI and observed whether JADI correlate with traditional measures or not.

## Materials and methods

The study population consisted of 49 consecutive patients with ERA who attended the outpatient clinic at a tertiary care referral hospital. ERA was defined according to the ILAR criteria [1]: onset of arthritis before the 16th birthday and persisting for at least 6 weeks; arthritis and enthesitis, or arthritis or enthesitis with at least 2 of (a) presence of or a history of sacroiliac joint tenderness and/or inflammatory lumbosacral pain, (b) presence of HLA-B27 antigen, (c) onset of arthritis in a male over 6 years of age, (d) acute (symptomatic) anterior uveitis (e) history of ankylosing spondylitis, enthesitis related arthritis, sacroiliitis with inflammatory bowel disease, Reiter's syndrome, or acute anterior uveitis in a first-degree relative [1]. By definition, patients with psoriasis or a history of psoriasis in the patient or first-degree relative, presence of IgM rheumatoid factor and systemic JIA are not included in ERA subtype [1]. Other inclusion criteria were age more than 5 years and disease duration of greater than one year. Same Rheumatologist (PKS) examined all patients. Onset of symptoms was taken as the date of onset of disease.

JADI scores were calculated as described previously [13]. The index is composed of articular (JADI-A) and extraarticular (JADI-E) damage. In the JADI-A, 36 joints or joint groups are assessed for the presence of damage: cervical spine, shoulders, elbows, wrists, individual metcarpophalangeal and proximal interphalangeal joints, hips, and knees; right and left temporomandibular joints, ankle and subtalar joints and metatarsophalangeal joint of each foot are considered as a single unit. The damage observed in each joint is scored as 1 in case of partial damage, or 2 in case of severe damage, ankylosis, or prosthesis. Contractures and other joint deformities are scored when they are completely explained by prior damage and are not due to active arthritis and are present for at least 6 months. For each joint, only the most severe lesions are scored. The maximum possible JADI-A score is 72.

For JADI-E, muscle atrophy, osteoporosis with fractures or vertebral collapse, avascular necrosis of bone, significant abnormality of the vertebral curve due to leg length discrepancy or hip contracture, significant leg length discrepancy or growth abnormality of a bone segment, striae rubrae, subcutaneous atrophy resulting from intraarticular corticosteroid injection, growth failure, pubertal delay, diabetes mellitus and amyloidosis are scored as 1 if present; ocular complications like cataract or other complications of uveitis were scored as 1 if present, 2 if surgery was required and 3 in case of legal blindness.

Abdominal fat pad analysis for amyloidosis was done if there was edema, anasraca, proteinuria or disproportionately increased ESR. Growth was assessed by plotting height and weight on standardized Indian pediatric growth charts. Growth failure was defined as the presence of two or more of the following: less then 3rd percentile height for age, growth velocity less than 3^rd ^percentile for age or crossing at least 2 centile on growth chart [13]. Delayed puberty was defined if no testicular enlargement occurred by 14 years of age.

Educational level achieved, loss of school years, functional status measured by HAQ-S [14] were also recorded. For the purposes of analysis, the HAQ-S score was divided into 4 categories: 0 = no disability, >0 and ≤ 0.5 = mild disability, >0.5 and ≤1.5 = moderate disability, and > 1.5 = severe disability [13]. Hundred mm Visual Analogue Scale (VAS) was used for parent/patient's assessment of their disease.

Examination included physician's global assessment on 100 mm VAS, number of active joints as defined by presence of swelling (excluding bony swelling) or any two of limitation of motion (LOM), pain, heat, or tenderness and number of joints with limited range of motion (ROM); 67 joints were assessed [13].

Enthesitis was defined as discretely localized tenderness at the point of insertion of ligaments, tendons, joint capsules, or fascia to bone [12,16]. Anterior lumbar flexion was assessed using the modified Schober's method [12,17]. Reduced lumbar flexion was defined as values ≤ 6.5 cm in boys and ≤ 5.5 cm in girls [11,18]. Inflammatory back pain was defined as lumbosacral spinal pain at rest, with morning stiffness that improved with movement [1,12]. Erythrocyte Sedimentation rate (ESR) was measured by Westergren method. Remission was defined as described previously [19].

### Statistical analysis

We used SPSS (version 13) software for statistical analysis. Spearman's rank correlation coefficient (rS) was used to assess correlations among different parameters. Correlations > 0.7 were considered high, 0.4 to 0.7 moderate and <0.4 low [13,20]. P values less than 0.05 were considered significant and less than 0.01 as highly significant.

## Results

The median age at the time of the study of the 49 patients with ERA was 18.0 years (range 10–27 years) and the median duration of disease was 6.0 years (1–17 years). All the patients were male and median age at disease onset was 11.0 (6–15) years. Half (53.1%) of the patients were HLA B 27 positive. Four (8.1%) patients were in remission at the time of the study. Twenty-eight patients (57.1%) had joints with limitation of motion with median of 2.0 joints (0–9); the frequency distribution of number of joints with limitation of motion is shown in table 1. Fourteen (28.6%) and 32 (65.3%) of the patients had decreased anterior lumbar flexion movement by modified Schober's method and inflammatory backache respectively. Active enthesitis was present in 62.59% of the patients.

**Table 1:** Frequency of number of joints with limited range of motion (LOM)

Number of joints with LOM	Frequency (N = 49)	Percentage
0	21	42.9

1	3	6.1

2	10	20.4

3	2	4.1

4	6	12.2

5	2	4.1

6	1	2.0

8	3	6.1

9	1	2.0

At the time of the study, 45 patients were on NSAID; Indomethacin was the most commonly prescribed NSAID- 25 patients (51.0%) were on it. Four (8.1%) patients were on oral steroid, in a dose of 7.5–10 mg daily. Eighteen patients (37.3%) had received intra-articular (IA) steroid. Nineteen patients were on DMARDs at the time of the study; Methotrexate was the commonest DMARD used (n = 9, 18.3%); 4 (8.3%) patients were on Salazopyrine. Side effects of drug were seen in 5 patients. Cytopenia occurred in 3 and GI toxicity in 2.

### JADI-A

In JADI-A scoring 17 patients (34.7%) had damaged joints. JADI-A score varied from 0 – 12 with a median of 1.0. Hip was the commonest damaged joint (n = 14; 28.6%), followed by ankles (7, 14.3%), cervical spine (3, 6.1%), elbows (2, 4.1%) and knees (1, 2.0%). None of the patients had temporomandibular, shoulder, wrist and MTP joint damage. Small joints of the hands were damaged in 2 (4.1%) patients. The frequency distribution of JADI-A score are shown in table 2.

**Table 2:** Frequency of juvenile arthritis damage index-articular (JADI-A)-score

JADI Score	Frequency (N = 49)	Percent
0	32	65.3

1	2	4.1

2	8	16.3

3	2	4.1

4	2	4.1

5	2	4.1

12	1	2.0

### JADI-E

Five (10.2%) patients had extraarticular damage with JADI-E. Three patients (6.1%) had severe muscular atrophy. Four (8.2%) patients had growth failure; pubertal delay was seen in 2 (4.1%). Secondary amyloidosis was seen in 1 (2.0%). None of the patients had ocular damage.

### Disease activity

Parent's/patient's global assessment on visual analogue scale (VAS) ranged from 0–100 mm (median 40.0); median score of physician's global assessment in VAS was 40.0 mm (range 0–90 mm). Correlation between the 2 global assessment was good with rS 0.73 (p < 0.01). Number of active joints varied from 0–9 (median 2.0). Duration of EMS ranged from nil to whole day (median nil) and Median ESR was 48.0 (7–140) mm/hour.

### Disability

There was no disability in 32.7% patients, mild disability in 18.4%, moderate disability in 34.7% and severe disability in 14.3%. Median HAQ-S was 1.0 (0–3).

### Loss of school years

Thirty two (65.3%) patients lost some years of education, with number of years lost due to disease ranging from 0–10 (median 1.0) years.

### Correlation of JADI with other disease variables

As shown in table 3, JADI correlated with HAQ-S, parent's or patient's global assessment, physician's global assessment, ESR, and radiological damage with p < 0.01. Correlation with HAQ-S, ESR, Patient's/parent's & physician's global assessment was modest and with JADI-E low.

**Table 3:** Correlation of JADI-A (2 tailed Spearman's correlation coefficient)

Parameter	Correlation coefficient (rS)
Physician's global assessment	0.691**

Radiological damage	0.664**

HAQ-S	0.526**

ESR	0.489**

Parent's/patient's global assessment	0.483**

JADI-E	0.311*

Active joint count	0.278

Duration of disease	0.147

Loss of education	0.1

DMARD use	0.046

Steroid use	-0.029

*p < 0.05; ** p < 0.01

HAQ-S had high correlation with limitation of spinal mobility by modified Schober's method (table 4). Correlation of JADI-A with limitation of spinal mobility was low. Presence of enthesitis has fair correlation with HAQ-S (p < 0.01) and JADI-A (0.01) (table 5).

**Table 4:** Correlation of limitation of spinal mobility by modified Schober's method (2 tailed Spearman's correlation coefficient)

Parameter	Correlation coefficient (rS)
Physician's global assessment	0.520**

Hip joint damage	0.437**

Knee joint damage	0.228

Ankle joint damage	0.150

JADI-A	0.334*

HAQ-S	0.701**

ESR	0.478**

Parent's/patient's global assessment	0.476**

JADI-E	-0.035

Active joint count	0.304*

Duration of disease	0.095

Loss of education	0.179

*p < 0.05; ** p < 0.01

**Table 5:** Correlation of presence of enthesitis with other measures of disease

Parameter	Correlation coefficient (rS)
Physician's global assessment	0.526**

Loss of spinal mobility	0.388**

Hip joint damage	0.313*

Knee joint damage	-0.189

Ankle joint damage	0.066

JADI-A	0.306*

HAQ-S	0.371**

ESR	0.309*

Parent's/patient's global assessment	0.409**

JADI-E	0.072

Active joint count	0.425**

Duration of disease	0.027

Loss of education	0.264

*p < 0.05; ** p < 0.01

## Discussion

Our study shows that more than half of the ERA patients have articular damage and one tenth have extraarticular damage at a median duration of disease of 5.0 years. Moderate to severe limitation of function was present in 49.0% patients. One third of the patients had limitation of movement of lumbar spine. More than 90% patients continued to have active disease.

More than 60% of our patients lost some years of education which is high compared to western literature, though most of previous studies included all JIA subtypes; only 3% of patients were behind age appropriate grade in a study from Canada [21]; education level attainment of adults with JIA was similar to controls in 2 studies from UK and Finland[22,23]. This could be related to poor social support system in a resource deficient country.

One third of the patients had damaged joints in JADI-A. Ours is the first study that has used JADI to assess outcome in patients with ERA. Hip was the commonest joint damaged in our study followed by knees, ankles and cervical spine. In contrast, in the previous study wrist, elbow and the interphalangeal joints were more often involved. This may be explained by the difference in subtypes of JIA patients between the two study groups: 60% of their patients had oligo articular disease and ERA patients were excluded from their study [13].

The median JADI-A score in our study is higher compared to the previous study [13] though the median duration of disease was comparable (5.0 Vs 7.3 years). This may be because numbers of patients on DMARD were fewer in our study as effective DMARD for ERA are limited except anti TNF which most of our patients cannot afford [24].

As with the previous study [13], JADI had moderate correlation with traditional outcome measures like HAQ-S, Patient's/parent's & physician's global assessment in our study. However, half of the patients had functional limitations though only 1/3^rd ^had damage in JADI-A score. The high degree of functional limitation with low JADI-A score may be due to under-representation of damage in lower limb joints in JADI score-midtarsal and subtarsal joints are not scored and MTP joints are clubbed together, which are commonly involved joints in ERA. For that reason, though more than half of the patients had damaged joints when 67 joints were counted in each patient compared to 1/3^rd ^in JADI-A score.

The finding of a relatively high level of functional limitaion in ERA is supported by a recent study demonstrating more functional impairment in juvenile AS than in adult AS [6]. The HAQ-S indices in our patients tended to be higher than the indices observed by previous authors [9,12,23]. The high degree of functional limitation and articular damage in our patients may also be due to selection bias as ours is a tertiary care and hospital and more severe cases may have been included.

Another limitation of application of JADI in ERA is absence of scoring for spinal involvement. Limitation of spinal mobility had high correlation with HAQ-S; but its correlation with JADI-A was modest. Inclusion of limitation of spinal movement in JADI may be helpful to detect damage in ERA. One third of our patients had limitation of lumbar spine movement by modified Schober's method, which is lower than the previous study [12]. Reduced spinal mobility occurs in three fourths of patients with ERA; half of them experience inflammatory back pain which is comparable to our study [12].

More than 60% of patients continued to have enthesitis which is not measured by JADI. Patients with persistent enthesitis develop severe functional and structural problems, including various degrees of enthesophytosis [24]. Presence of enthesitis had a highly significant correlation with HAQ-S in our study; but it had no correlation with JADI-E.

Other extraarticular damage i.e. JADI-E was uncommon in our study. Frequency of ophthalmological complication in ERA are less than other subtypes specially oligoarticular JIA. Since ERA patients usually have acute anterior uveitis which seldom leaves significant residua even if recurrent [25]. Frequency of amyloidosis in our study is comparable to an earlier study by Pkham et al [26]. For growth failure most of the previous outcome studies on JIA didn't separate subtypes; frequency of 7.8% in our study is comparable to the study by Viola s et al [13].

The remission rate among our patients with ERA was lower than that reported by other authors [8,12]. The frequency of remission in juvenile spondylarthropathy, has ranged from 17% to 37% [7,9]. This differences may be due to different definition of remission in various studies. The remission criteria used in our study have been evaluated for oligoarthritis and polyarthritis but not for ERA [19]. Patients needing NSAID were not defined as having remission in our study; some may argue against it as patients not needing DMARD may be defined as having in remission if they otherwise fullfil remission criteria.

Our study has many limitations: numbers of patients are small, is a cross sectional study, the average disease duration is only 5 years and lacks radiological outcome

In conclusion, three fourth of the ERA patients have functional limitations. JADI is a useful tool to measure articular and extraarticular damage in ERA; it has a good correlation with traditional outcome measures. However, it underestimate lower limb joint damage and does not estimate spinal damage as well as enthesitis. Inclusion of limitation of movement of spine, foot joints and score for enthesitis in JADI may increase its usefulness in ERA.

## Competing interests

The authors declare that they have no competing interests.

## Authors' contributions

PS collected the data, wrote and revised the manuscript. RNM helped with planning the study, writing and revising the manuscript. AA conceived and planned the study, helped with analysis of data, writing and revison of the manuscript. All authors read and approved the final version of the manuscript.
